# Identification of *Streptomyces* spp. from garbage dump soils in Surabaya, Indonesia

**DOI:** 10.14202/vetworld.2022.634-639

**Published:** 2022-03-22

**Authors:** R. Kurnijasanti, S. A. Sudjarwo

**Affiliations:** Department of Pharmacology, Faculty of Veterinary Medicine, Universitas Airlangga, Surabaya, Indonesia

**Keywords:** identification, infectious disease, the garbage dump soil, the new type of *Streptomyces*

## Abstract

**Background and Aim:**

*Streptomyces* is a well-known agent of secondary metabolite production. This study aimed to identify *Streptomyces* spp. from garbage dump soils in Surabaya based on the *16S rRNA* gene sequence. Moreover, the structure of new chemical compounds used for treating infectious diseases in humans, animals, and plants was elucidated.

**Materials and Methods:**

We isolated *Streptomyce*s spp. from garbage dump soils in Surabaya. In this study, all isolates were characterized according to phenotype, and they were also confirmed by *16S rRNA* gene sequence analysis using real-time polymerase chain reaction. Multiple sequence alignment and molecular phylogeny analyses were conducted using the MEGA 5.0 software, and then the TREE VIEW program was used to display the phylogenetic tree. The level of DNA similarity was also evaluated using the basic local alignment search tool (BLAST) program and then compared with nucleotide sequences stored in the GenBank database using National Center for Biotechnology Information BLAST.

**Results:**

The eight *Streptomyces* spp. showed different nucleotide sequence lengths in gel electrophoresis and photography, which is in accordance with the results observed in the phylogenetic tree. New types of *Streptomyces* spp., *Sp-*C, *Sp-*D, *Sp-E*p, *Sp-*G, and *Sp-I*, were found from the waste heap in Surabaya. Of these, *Sp-Ep* was very closely related to *Streptomyces indonesiasis* and *Streptomyces* *nashvillensis*. *Sp-F* was identified as *Streptomyces levis* strain *NRRL B-24299*, and *Sp-C* was identified as *Synodontis filamentosus*. *Sp-D* was related to *Sida javensis* and *Staphylococcus roseus*. *Sp-G* was related to *Streptomyces roseoviridis* strain *NBRC 12911* and *Streptomyces thermocarboxydovorans* strain AT52. *Sp-I* was related to *Streptomyces cangkringensis* and *Streptomyces asiaticus*. Finally, *Sp-A* was related to *Sansevieria laurentii* strain *LMG 19959*.

**Conclusion:**

Based on the phylogenetic tree, new strains of *Streptomyces* isolate, namely, *Sp-D*, *Sp-Ep, Sp-G, and Sp-I*, were found in the garbage dump soils of Surabaya. This new strain can produce antibiotics to be used as an alternative to antibiotics; however, further research is needed to confirm the activity.

## Introduction

The genus *Streptomyces* is widely used in the production of secondary metabolites, such as antibiotics, antifungal, antiparasitic, and anticancer agents, possessing diverse biological activities [1,2]. Most *Streptomyces* spp. produce various antibiotics such as aminoglycosides, glycopeptides, anthracyclines, macrolides, nucleosides, β-lactams, peptides, polyenes, polyethers, and tetracyclines. *Streptomyces* spp. produce approximately 75% of antibiotics that are used both clinically and commercially [3,4]. Various biodegradative and biotechnological screening processes, based on the diversity of actinomycetes, especially *Streptomyces*, are applied in the pharmaceutical industry. Moreover, several new antibiotics with numerous variations have been produced using *Streptomyces* spp., which exceed those produced using other genera of actinomycetes. A large number of species or strains of *Streptomyces* have been demonstrated to produce new antibiotics existing in nature. More than 6000 compounds have been obtained from *Streptomyces* spp., which are commercially available as antibiotic, antiparasitic, antifungal, and anticancer agents. One species of *Streptomyces* can produce two to three natural antibiotics. This is accomplished by isolating and characterizing tens of thousands of these compounds. The majority of them have been developed into drugs that can be used to treat various types of diseases in humans, animals, and plants [2,5,6].

*Streptomyces* spp. have been identified primarily using conventional classification methods based on their morphological and phenotypic characteristics. The impact on the taxonomy of *Streptomyces* increased over the past few decades due to the use of molecular biology methods, such as *16S rRNA* gene sequencing andBOX-polymerase chain reaction (PCR) fingerprinting [7-9].

In the present study, we used *16S rRNA* gene sequencing to classify *Streptomyces* isolates from garbage dump soils in Surabaya, Indonesia.

## Materials and Methods

### Ethical approval

This study did not use any experimental animals. Hence, ethical approval did not require in this study.

### Study period and location

This research was conducted from January 2021 to August 2021. Isolation and identification of Streptomyces were carried out at the Tropical Diseases Center, Airlangga University.

### Isolation of *Streptomyces* spp. from garbage dump soil samples

The garbage dump soil sample collected from Surabaya, Indonesia, was used for the isolation of *Streptomyces* spp. Briefly, 1 g of the soil sample was transferred into a flask containing 10 mL distilled water. Then, it was filtered through a two-layered muslin cloth. The sample was diluted to 10^–3^, 10^–4^, and 10^–5^ concentrations. Next, 0.2 mL of each dilution was placed on starch agar medium (starch 9.0 g, l-asparagine 9.0 g, ammonium sulfate 2.0 g, Tris 2.0 g, sodium chloride 1.0 g, dipotassium sulfate 0.5 g, magnesium sulfate 0.2 g, calcium chloride 0.1 g, trace solution 1 mL, potassium dihydrogen phosphate 0.5 g, and agar 15 g, all of which were dissolved in 1 L distilled water at pH 7.0) plates, supplemented with the antifungal agent nystatin (50 μg mL^–1^), and incubated for 7 days at 35±20°C. Plates with approximately 200 colonies were selected. Single colonies were streaked on the same medium to purify selected colonies.

### Total DNA isolation

Khattab *et al*. [10] reported that molecular and bioinformatics analyses were conducted to identify *Streptomyces* strains. Genomic DNA was extracted using the Corbin method with several modifications, according to a previously described protocol [10,11]. Briefly, one colony was cultured in 50 mL of liquid ISP4 medium at 28°C in a shaking incubator for 18-24 h. Then, the culture was centrifuged at 5000 rpm for 3 min, and the resulting supernatant was discarded. Then, Streptomyces were collected, by being suspended in Solution I containing 1 mM ethylenediaminetetraacetic acid, 0.5% sodium dodecyl sulfate 10 mM Tris (pH 7.4), and 0.1 mg/mL proteinase K. Furthermore, the streptomyces suspension was added to Solution II containing 0.8 M NaCl and 1% CTAB for 1 h at 37°C, it will be added to the lysate, and then incubated for 20 min at 65°C. The sample was extracted using chloroform: isoamyl alcohol with the same volume (24:1). The nucleic acid is precipitated in the aqueous phase with isopropanol and then purified using 70% ethanol.

### Amplification and sequencing of *16S rRNA* gene by PCR

The primers Strep F; 5-AGAGTTTGAT CCTGKGTCAG-3 and Strep R; 5-AAGGGAG GTGATCCAKKGKGA-3 were used in PCR amplification of the *16S rRNA* gene against *Streptomyces* strains [12-14]. Each primer of the PCR mixture in 50 I polymerase buffer contained 30 pmol, 100 ng of chromosomal DNA, 200 M dNTPs, and 2.5 U of Taq polymerase. The primary denaturation temperature for PCR amplification was 94°C for 1 min, followed by 94°C for 1 min, and the annealing temperature was 57°C for 60 s. The extension step consisted of 35 cycles of 72°C for 60 s. The final extension was performed at 72°C for 5 min. Then agarose gel electrophoresis was used to analyze the PCR reaction mixture, which is a size marker. In addition, it is indicated by the use of Nucleic Acid Gel Electrophoresis and Blotting (Thermo Scientific™ Fermentas GeneRuler DNA Ladder Mix, USA) 1 kb. The remaining mixture was purified using QIA rapid PCR purification reagent (Qiagen, USA). The Terminator Cycle Sequencing kit was used to obtain the *16S rRNA* gene sequence of both strands (ABI Prism 310 Genetic Analyzer, Applied Biosystems, USA).

### Gel electrophoresis and photography

To separate the PCR amplification products, the mini-gel set (Bio-Rad, USA) was used to process 1% w/v ultrapure agarose powder in 1× TBE buffer (pH 8.3) at 100 V for 60-70 min. The gel was stained with ethidium bromide (0.5 g/mL) and then analyzed using BioDocAnalyze (Biometra, Germany). A molecular weight marker of 250 bp was used.

### Sequence similarities and phylogenetic analysis

Multiple sequence alignment and molecular phylogeny analyses were performed using the MEGA 5.0 software (www.megasoftware.net). The TREE VIEW program was used to display the phylogenetic tree [15-17]. The level of DNA similarity was evaluated using the basic local alignment search tool (BLAST) program (www.ncbi.nlm.nih.gov/blst) by comparing with nucleotide sequences stored in the GenBank database using National Center for Biotechnology Information (NCBI) Blast [18,19].

### Identification of *Streptomyces* isolates

This study applied a numerical taxonomy using the 16S rRNA-based *Streptomyces* spp. program for species identification. This was done as recommended in the international key [9,13].

## Results

### Agarose gel electrophoresis of PCR amplification products of genomic DNA of *Streptomyces* spp.

**Figure-1:**
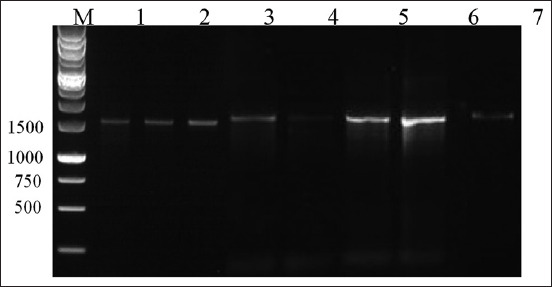
The electrophoresis of agarose gel polymerase chain reaction amplification product of *Streptomyces* spp. genomic DNA isolated from landfill soil in Surabaya. Lane M: Marker, Lane A: *Streptomyces* *Sp-A*, Lane C: *Streptomyces* *Sp-C*, Lane D: *Streptomyces* *Sp-D*, Lane Ea: *Streptomyces* *Sp-Ea*, Lane Ep: *Streptomyces* *Sp-Ep*, Lane F: *Streptomyces* *Sp-F*, Lane G: *Streptomyces* *Sp-G*, Lane I: *Streptomyces* *Sp-I*.

Different nucleotide sequence lengths were detected in the sequencing results of the eight *Streptomyces* spp. from the garbage dump soils of Surabaya, Indonesia. *Sp-A* and *Sp-Ea* showed a nucleotide sequence length of 1000 bp. *Sp-C, Sp-F, Sp-G*, and *Sp-I* showed a nucleotide sequence length of 1250 bp. *Sp-D* showed a nucleotide sequence length of 1200 bp. *Sp-Ep* showed the shortest nucleotide sequence length of 750 bp. Electropherogram images (Thermo Fisher Scientific, USA) and nucleotide sequence data of *Sp-A*, *Sp-C*, *Sp-D*, *Sp-Ea*, *Sp-Ep*, *Sp-F*, *Sp-G*, and *Sp-I* were obtained. As shown in Figure-1, *Sp-A*, *Sp-C*, *Sp-D*, *Sp-Ea*, *Sp-Ep*, *Sp-F*, *Sp-G*, and *Sp-I* produced bands with a dominant size of approximately 1500 bp. This size of the bands was confirmed using the 16S rRNA gene, that is, 1500 bp, and the subsequent bands were sequenced using the automatic ABI Prism 310 method.

### Molecular identification of the isolated *Streptomycetes*

The *16S rRNA* gene was used as a reference for bacterial identification because this gene is the most resistant to change or evolution. Table-1 shows the results of the gene sequencing of *16S rRNA* using BLAST, wherein the eight strains were confirmed as *Streptomyces* spp. The analysis was based on partial *16S rRNA* gene sequencing and the nucleotide sequence data stored in the GenBank database (NCBI database).

**Table-1:** Analysis of streptomycete population clusters isolated from desert and savanna ecosystems in Sudan and identified based on 16S rRNA gene analysis.

Streptomyces isolate	Identification	Percentage of identification accuracy/similarity (%)
*Sp-A*	*Streptomyces polychromogenes* subsp. *areniculus* gene for 16S rRNA, partial sequence, strain: *NBRC 13872*	95
*Sp-C*	*Streptomyces polychromogenes* subsp. *arenicolus* gene for 16S rRNA, partial sequence, strain: *NBRC 13872*	97
*Sp-D*	Streptomyces spp. 172618 16S ribosomal RNA gene, partial sequence	97
*Sp-Ea*	*Streptomyces* spp. ACT-0095 16S ribosomal RNA gene, partial sequence	96
*Sp-Ep*	*Streptomyces* spp. ACT-0095 16S ribosomal RNA gene, partial sequence	99
*Sp-F*	*Streptomyces polychromogenes* subsp. *arenicolus* gene for 16S rRNA, partial sequence, strain: *NBRC 13872*	99
*Sp-G*	*Streptomyces polychromogenes* subsp. *arenicolus* gene for 16S rRNA, partial sequence, strain: *NBRC 13872*	98
*Sp-I*	*Streptomyces polychromogenes* subsp. *arenicolus* gene for 16S rRNA, partial sequence, strain: *NBRC 13872*	98

The nucleotide sequence homology of *Streptomyces* spp. from the garbage dump soils of Surabaya showed a sequence similarity of 95-99% with the GenBank database sequences. The similarity values of the nucleotide sequences were as follows: 95% for *Sp-A*, 97% for *Sp-C* and *Sp-D*, 96% for *Sp-Ea*, 99% for *Sp-Ep* and *Sp-F*, and 98% for *Sp-G* and *Sp-I*. Table-1 presents an overview of the homology results derived from the nucleotide sequences of *Streptomyces* spp. RKBS soil isolates with the nucleotide sequences of *16S rRNA* gene in the NCBI database.

*Sp-C, Sp-F, Sp-G*, and *Sp-I* showed the highest similarity with the *Streptomyces* *polychromogenes* subsp. *arenicolus* strain *NBRC 13872*. The nucleotide sequences of *Sp-Ea* and *Sp-Ep* showed the highest similarity with *Streptomyces* spp. *ACT-0095*. Those of *Sp-D* showed the highest similarity with *Streptomyces* spp. 172618.

### Phylogenetic relationships based on *16S rRNA* sequences of *Streptomyces* spp.

**Figure-2:**
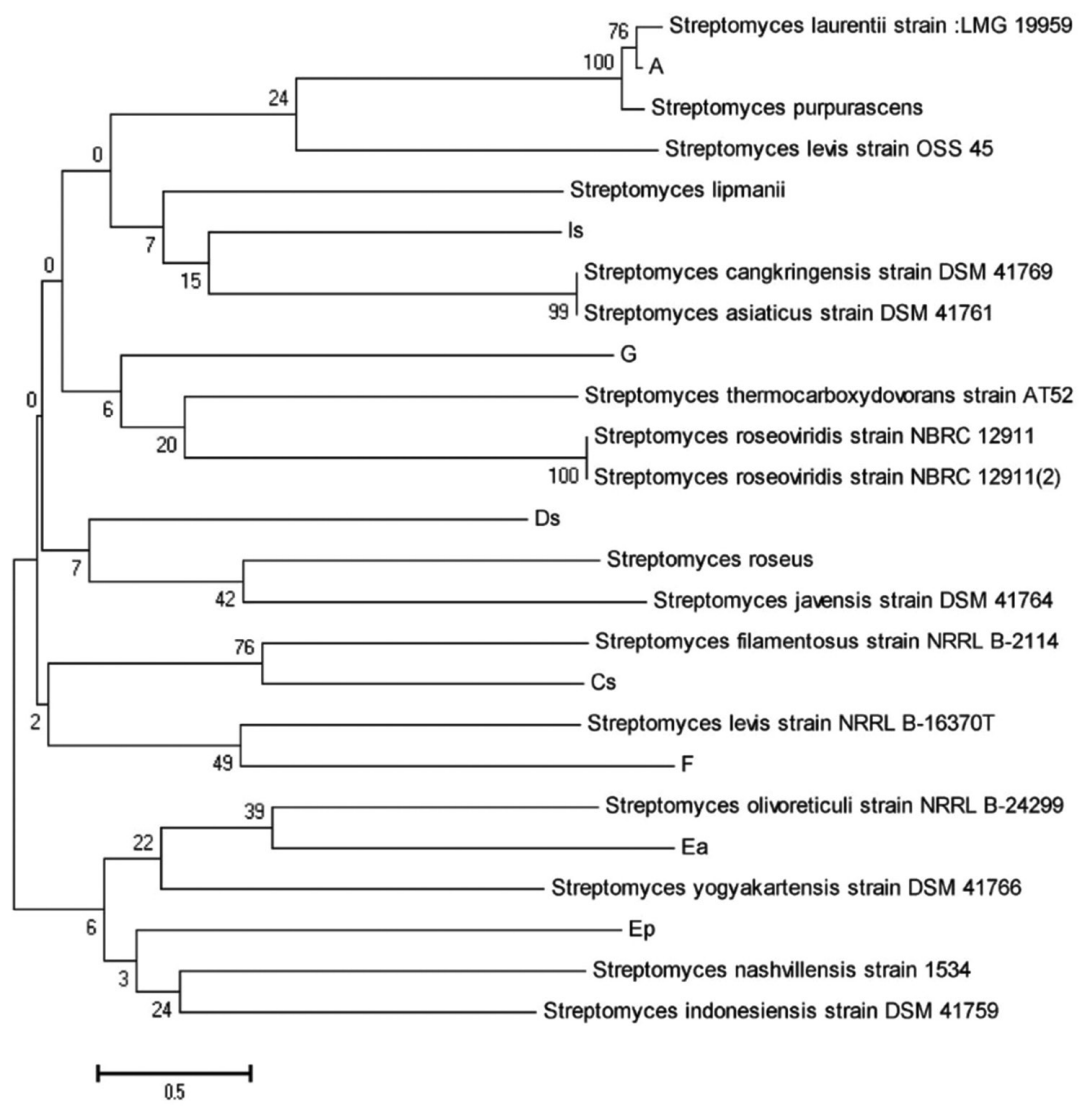
Phylogenetic relationships based on 16S rRNA sequences among 8 Streptomyces strains in relation to closely related validly described species. Evolutionary analysis was based on the Neighbor-joining method using MEGA X software. The bar represents 0.005 nucleotide substitutions per alignment position; numbers above the branches are bootstrap values.

The identification of *Streptomyces* spp. was based on the 16S rDNA sequence data, which provided the best information about *Streptomyces* and can be used to identify several new types of *Streptomyces* strains. The phylogenetic tree shown in Figure-2 was derived using the neighbor-joining method from the distance matrix. The majority of sequences clustered into groups in the phylogenetic analysis. However, the results revealed the presence of different types of *Streptomyces* 16S rDNA sequences, suggesting several new types of *Streptomyces* species.

The nucleotide sequence of the *16S rRNA* gene from *Streptomyces* spp. was used as the basis for constructing the phylogenetic tree, wherein the sequencing results were obtained using the MEGA version 5.0 program. Next, the eight *Streptomyces* spp. from the garbage dump soil isolates were compared with five local Indonesian isolates of *Streptomyces* spp. and 12 *Streptomyces* isolates from the GenBank database.

Figure-2 shows the comparison of sequencing results of the different *Streptomyces* isolates and the relationship between the isolates and their closest phylogenetic neighbors. The different phylogenetic lines were formed from several sequences, whereas the groups in the *Streptomyces 16S rRNA* gene tree contained those from other sequence groups. The phylogenetic tree analysis revealed the presence of a new type of *Streptomyces* spp., namely, *Sp-Ep*, which was still related to *Streptomyces indonesiasis* and *Streptomyces nashvillensis*. Moreover, researchers observed that *Streptomyces olivoreticuli* was related to *Streptomyces yogyakartensis*, which was termed as *Sp-Ea*. Another type identified in this study was *Streptomyces levis* strain *NRRL B-24299*, which was termed as *Sp-F*. *Synodontis filamentosus* was termed as *Sp-C*. Another new type of *Streptomyces* strain closely related to *Sida javensis* and *Staphylococcus roseus* was termed as *Sp-D*. The type *Sp-G* was related to *Streptomyces roseoviridis* strain *NBRC 12911* and *Streptomyces thermocarboxydovorans* strain *AT52*. The next new type *Sp-I* was related to *Streptomyces asiaticus* and *Streptomyces cangkringensis*. Finally, the type *Sp-A* was related to *Streptomyces* *LMG 19959*. In general, all these isolates belong to the same genus, namely, *Streptomyces*, but they are of different types.

As shown in Figure-2, the phylogenetic relationships based on the order of *16S rRNA* between the eight *Streptomyces* are interrelated between one species and another. Furthermore, the neighbor-joining method using MEGA X software (www.megasoftware.net) was used as the basis for evolutionary analysis. 0.005 nucleotide substitutions per alignment position were represented by stems, and the number above that branch was called the bootstrap value.

## Discussion

In the present study, we used *16S rRNA* gene sequencing to classify *Streptomyces* isolates from garbage dump soils in Surabaya, Indonesia and identified new, specific strains that can produce antibiotics to be used as alternative drugs. *Streptomyces* represent an important source of bioactive compounds, which are widely used commercially to produce antibiotic, antiparasitic, antifungal, and anticancer agents [2,4,6]. Furthermore, continuing the search for new bioactive compounds is important because of the increasing number of antibiotic-resistant bacteria every year. However, huge challenges exist in this regard because identifying new secondary metabolites is extremely difficult, which thus requires the isolation, characterization, and screening of new members of the genus *Streptomyces*. Moreover, several newly confirmed bioactive compounds are derived from *Streptomyces* from unexplored habitats, which might be extremely rich sources of antibiotics. Therefore, we isolated *Streptomyces* spp. from the garbage disposal soils of Surabaya, Indonesia.

The *16S rDNA* gene was amplified using primers to identify *Streptomyces* isolates, followed by PCR for molecular identification, which is a sensitive and specific detection method for *Streptomyces*. The 16S rDNA target gene was used for the selected PCR primer for detecting the eight *Streptomyces* isolates. The BLAST was used to compare the *Streptomyces 16S rRNA* gene sequences and those in public databases, consistent with that recommended in the NCBI website [6,12,19]. This was done to determine the similarity between sequences in the GenBank database.

In the phylogenetic tree construction for *Sp-A*, *Sp-C*, *Sp-D*, *Sp-Ea*, *Sp-Ep*, *Sp-F*, *Sp-G*, and *Sp-I* strains, the *16S rRNA* gene sequences with high similarity were used in this study. The sequencing results showed that the strains belonged to the genus *Streptomyces*. They were used to compare several strains described validly with local Indonesian isolates that were selected as outgroups. These isolates were closely related to several strains, including *Sansevieria laurentii LMG 19959*, *Sphenarium purpurascens*, *Streptomyces lewis OSS 45*, *Streptomyces lipmanii*, *S. cangkringensis DSM 41761*, *S. thermocarboxydovorans AT 52*, *S. javensis DSM 41764*, *S. roseoviridis NBRC 12911*, *S. roseus*, *S. lewis NRRL B-16370T*, *S. filamentosus NRRL B-2114*, *S. olivoreticuli NRRL B-24299*, *S. yogyakartensis DSM 41766*, *S. nashvillensis 1555534*, and *S. indonesiasis* *DSM 41759*. The *Streptomyces* type *Sp-Ep* identified in this study was closely related to *S. indonesiasis* and *S. nashvillensis*. *Sp-Ea* was related to *S. olivoreticuli* and *S. yogyakartensis*. *Sp-F* was related to *S. levis* strain *NRRL B-24 299*. *Sp-C* was related to *S. filamentosus*. *Sp-D* was related to *S. javensis* and *S. roseus*. *Sp-G* was closely related to *S. roseoviridis* strain *NBRC12911* and *S. thermocarboxydovorans* strain *AT52*. Meanwhile, another new species *Sp-I* was closely related to *S. cangkringensis* and *S. asiaticus*. Finally, *Sp-A* was found to be related to *S. laurentii* strain *LMG 19959*. All isolates identified in this study belonged to the same genus *Streptomyces* and were distinguished by their type. Complete information is presented in the phylogenetic tree of *Streptomyces* spp.(Figure-2).

## Conclusion

The phylogenetic tree analysis of *Streptomyces* spp. revealed the presence of the new types of *Streptomyces* species in Surabaya garbage dump soils: *Sp-D*, *Sp-Ep*, *Sp-G*, and *Sp-I*. This new strain can produce antibiotics to be used as an alternative to antibiotics; however, further research is needed to confirm the activity.

## Authors’ Contributions

RK and SAS: Designed and conceptualized the study. RK: Conducted the study, analyzed the results, and literature search. SAS: Supervised the study and drafted the manuscript. SAS and RK: Revised the manuscript. All authors read and approved the final manuscript.
